# Objectivity for the research worker

**DOI:** 10.1007/s13194-021-00400-6

**Published:** 2021-09-08

**Authors:** Noah van Dongen, Michał Sikorski

**Affiliations:** 1 University of Amsterdam, Amsterdam, The Netherlands; 2 University of Gdańsk, Gdańsk, Poland; 3 Warsaw University of Technology, Warsaw, Poland

**Keywords:** Objectivity, Bias, False-positive rate, Replication crisis

## Abstract

In the last decade, many problematic cases of scientific conduct have been diagnosed; some of which involve outright fraud (e.g., Stapel, 2012) others are more subtle (e.g., supposed evidence of extrasensory perception; Bem, 2011). These and similar problems can be interpreted as caused by lack of scientific objectivity. The current philosophical theories of objectivity do not provide scientists with conceptualizations that can be effectively put into practice in remedying these issues. We propose a novel way of thinking about objectivity for individual scientists; a negative and dynamic approach.We provide a philosophical conceptualization of objectivity that is informed by empirical research. In particular, it is our intention to take the first steps in providing an empirically and methodologically informed inventory of factors that impair the scientific practice. The inventory will be compiled into a negative conceptualization (i.e., what is not objective), which could in principle be used by individual scientists to assess (deviations from) objectivity of scientific practice. We propose a preliminary outline of a usable and testable instrument for indicating the objectivity of scientific practice.

*The first principle is that you must not fool yourself, and you are the easiest person to fool.* Richard Feynman (Cargo Cult Science, 1974)

## Introduction: a story about a scientist

Despite its undeniable success (e.g., electricity, space flights, etc.), science seems to be in a difficult position today. In the last few years, many problematic cases of scientific conduct were diagnosed, some of which involve outright fraud (e.g., Stapel, 2012) while others are more subtle (e.g., supposed evidence for precognition; Bem, 2011). These particular issues and the general lack of replicability of scientific findings (e.g., Open Science Collaboration, 2015) have contributed to what has become known as the *replication crisis* (e.g., Harris, 2017). In addition, the general public has become aware of these problems, which has shaken the general trust in science (e.g., Lilienfeld, 2012, Pashler & Wagenmakers, 2012, Anvari & Lakens, 2018).

Let us imagine a scientist, Dr. Jane Summers. Dr. Summers^1^ does research in cognitive psychology. One day, she reads about the low replicablity rate of the results of psychological studies (e.g., Open Science Collaboration, 2015). She becomes very concerned about the value of scientific results in general and her own research in particular. As a result, she is resolved to investigate to what extent her work is at risk of irreplicability and to ensure that her current and future work is as resilient as possible against such a fate. She decides that, apart from ensuring the accuracy and precision of her measurements, the methods she employs should not be significantly influenced by her feelings, values, biases and other idiosyncrasies. To her, this would mean that they are objective (Hawkins & Nosek, 2012; Ziman, 1996; Stegenga, 2011).^2^ Objectivity can be attributed, among others, to scientific measurements, tools for development/improvement of scientific theories, and/or to true-to-nature explanations. It ensures that study outcomes are not biased (e.g., over estimation of drug efficacy, under estimation of risk; Goldacre, 2014), positive research results are not false-positives (to a larger proportion than is allowed by the statistical method; Simmons et al., 2011), and are independently reproducible by other scientists (Simons, 2014; Lindsay, 2015; Altmejd et al., 2019; van Bavel et al., 2016). Dr. Summers considers objectivity to be essential to science^3^ and its absence to be a cause of the crisis that threatens the foundations of her research field. In short, Dr. Summers considers the assessment and safeguarding of scientific objectivity as being of vital importance. Plausibly, such sentiment toward objectivity is common among actual scientists. For example, we can easily imagine Prof. Bem wanting to present results as solid and close to incontrovertible evidence in favor of precognition as possible (Bem, 2011). Specifically, ensuring objectivity of his experiments would ensure that his claims are on solid ground.

It is therefore somewhat puzzling to Dr. Summers that a proper explication of objectivity appears to be lacking in science. She is unable to find tools for the qualitative and/or quantitative assessment of objectivity. Methodological reforms are inspired by problematic cases, for instance, measurement or impossible results (e.g., precognition; Bennett et al., 2010) or failures to reproduce established experimental results (e.g., Klein et al., 2018), rather than a clear understanding of objectivity. She could attempt to replicate her own work, have it replicated by others, and/or review her publication with respect to guidelines of statistical methods (see for instance Gervais, 2017, Carney, 2016) and, as a result, declare a lack of confidence in her own work (see for instance Rohrer et al., 2018), but nothing more systematic is available. Similarly, Prof. Bem would have a hard time providing an objectivity assessment of his precognition experiments with the currently available tools. Thus, Dr. Summers realizes that science could greatly benefit from having a definition of ‘objectivity’ that can be explicated in a quantitative or qualitative assessment of scientific practice.

Dr. Summers has a hunch that philosophy might be of assistance in defining objectivity. After a short review of the philosophical literature, she does not manage to find a notion of objectivity ready for use in scientific practice. Typically, such proposals are descriptive and therefore lacks a guiding force, because they are not supported by normative considerations. Other proposals are difficult or impossible to test, thus prohibiting scientists from assessing objectivity (Section 2). In effect, Dr. Summers becomes disheartened and contemplates quitting her quest for objectivity.

It is our opinion that we, philosophers, should not disappoint scientists like Dr. Summers in this respect and that philosophy can and should do better. We believe that the philosophical literature currently lacks a scientifically useful conceptualization of objectivity and we intend to fill this gap. In this article, we present a conceptualization of objectivity of scientific practice that is practicable by the individual scientist. We understand scientific practice as pertaining to empirical research, which include all activities done by scientist essential for this endeavor. These include study design, data collection and measurement, data analysis, result reporting etc.^4^ We recognize that the social and cultural conditions play a role in, for instance, determining what kind of research gets funded, and recognize the value of social epistemology and literature on non-epistemic values (e.g., Biddle, 2007, Bueter, 2015, Longino, 1990, Elliott and McKaughan, 2009). However, much of this is beyond the purview of what an individual researcher can control and therefore beyond the scope of our paper.^5^

Our aim is to provide a scientifically useful notion of objectivity. In order to be useful such a conceptualization must be both based on normative considerations and testable. For if it is not based on normative and reliable methodological results it is not clear if it possesses any guiding force and if it is not testable, it cannot be used to assess the objectivity of a given practice. In the next section, we will briefly discuss several philosophical views on objectivity and highlight where there is room for improvement. In the third section, we present a novel version of a negative approach to scientific objectivity and provide a testable conceptualization of objectivity that is based on robust empirical results and methodological considerations. Finally, we defend the fruitfulness of our notion by demonstrating how it can be used in scientific practice (Section 4.2) and provide a sketch of a tool for assessing objectivity inspired by our new conceptualization (Appendix A).

## Philosophy on objectivity

In philosophy of science, scientific objectivity is a well discussed notion. Following Reiss and Sprenger (2017), we can list three main ways of conceptualizing it. Firstly, objectivity can be understood as a faithfulness to facts. Secondly, something can be understood as objective when it is free from value commitments. Thirdly, objectivity can be understood as being free from scientists’ personal biases. Recently, proposals which have gained much popularity are pluralist notions of objectivity (e.g., Douglas, 2004, Megill, 1994, Wright, 2018). Such notions encompass some or all of mentioned individual notions (e.g., the value-free objectivity, value neutral objectivity, procedural objectivity etc.). Finally, there are negative conceptions of objectivity (e.g., Koskinen, 2020, Daston & Galison, 2010, Hacking, 2015) which claim that the objectivity consist of the absence of certain factors. In the case of (Daston & Galison, 2010), these are factors of scientific subjectivity which are recognized by the scientific community as particularly troubling or important in a given time period. In the case of Koskinen (2020), these factors are epistemic risks which arise from the imperfections of epistemic agents.

Despite this effort, it seems that a conceptualization of scientific objectivity that can be easily used by scientists has not yet been proposed. The literature is comprised of proposals that were not designed to fulfill such a practical role. Instead, they were designed to describe how the concept is used. Following Searle (1975) we will understand the difference between descriptive and normative discourse in terms of the direction of fit. The descriptive claims aim at describing reality (e.g., ‘there is no poverty in the world’). In contrast, normative claims are not descriptions of how things are, they are intended to describe how the word should be (e.g, ‘there should not be poverty in the world’). In other words, descriptive claims have *language-to-reality direction of fit* while normative claims have a *reality-to-language direction of fit*. Consequently, a descriptive theory of a given concept describes in precise terms (the meaning of) the concept, which is actually used by natural language speakers (or some sub-group of them). Such theories can be assessed empirically by comparing it with the intuitions of a target group. The examples of such theories are the semantics for conditionals (see e.g., Douven et al., 2018). On the other hand, a normative theory of a given concept presents (a meaning of) the concept which, when used, will be beneficial for the hypothetical users. For example, some of the formal theories on truth offer replacements for the concept of ‘truth’ used in natural language (see e.g., Scharp, 2013, Tarski, 1936). Authors of these proposals argue that new concepts are superior to the concept present in natural language, because, for example, they are not susceptible to notorious semantic paradoxes. In our article, we are interested in a normative theory of objectivity. Hence, we are less concerned with how the new conceptualization corresponds to how objectivity is used in natural language and more concerned with how the conceptualization promotes the methodological quality of science (e.g., replicability, lack of bias, etc.) and its results (e.g., approximately true / highly corroborated theories, theories with high predictive accuracy).

In light of the conflicting intuitions and conceptual confusion surrounding objectivity, the descriptive conceptualizations of objectivity are clearly useful. However, it is distinct from our aim of formalizing a notion that is normatively useful. Due to their descriptive aim, it is not clear if these theories can fulfill the normative task of guiding scientific practice and it would not be fair to assess them in this context. Some authors are explicit about the descriptive nature of their proposals. For example, the aims of Heather Douglas (2004) famous article seems to be primarily^6^ descriptive:

In this paper, I will lay out a complex mapping of the senses of objectivity. This mapping will make two contributions to current discussions. First, it will dissect objectivity along operationally distinct modes.[...] Second, the mapping will allow me to cogently argue that the different meanings of objectivity I explore here are not logically reducible to one core meaning. (Douglas, 2004, p. 454-455)

Similarly, Koskinen (2020) is explicit about the descriptive aim of her proposal:

In this article I defend a risk account of scientific objectivity. The account is meant to be a largely descriptive or even a semantic one; my aim is to draw together ideas presented in recent discussions, and to clarify what we philosophers of science do when we identify distinct, applicable senses of objectivity or call something objective. (Koskinen, 2020, p.1)

These quotes indicate that Douglas (2004) collects applicable notions of objectivity (*procedural objectivity, value free objectivity*, etc.) while Koskinen unifies those distinct meanings. Their aims are descriptive. In the case of other proposals, it is clear that they are descriptive due to their methodological approach. For instance, Datson and Galison’s (2010) historical methodology makes it a descriptive proposal.

Secondly, some of the proposed normative conceptualizations of objectivity are not suitable to be used by scientists. Such notions need to be testable. Otherwise, how can we assess if given scientific practice is objective or not? An example of a notion that fails in this respect is *value-free objectivity*. Value-free objectivity is based on a more general *value-free ideal*. The value-free ideal claims that scientists should not use their non-epistemic values, like ‘equality’ of ‘fairness’, when they justify their claims (e.g., Betz, 2013). This conception of objectivity claims that a scientific justification is objective as long as it is not influenced by non-epistemic values. There might be reasons to believe that value-free ideal should be followed (e.g., Betz, 2013, Sober, 2007) or that the corresponding notion of objectivity is compelling. However, many problems of value-free objectivity have been diagnosed. For instance, (Douglas, 2004), after (Rudner, 1953), argued that value free-ideal is unrealizable. Similarly, (Longino, 1996) claims that the distinction between epistemic and non-epistemic values, on which value-free objectivity is based, is ill-defined, making this conceptualization of objectivity problematic. Additionally, there are clear difficulties in assessing the value-free objectivity of scientific practice. Most glaringly, we do not have access to scientists’ intentions, thus we cannot judge what motivated their decisions and actions. Therefore, we cannot use a theory which defines objectivity in terms of values used by scientist to, for example, assess the objectivity of a procedure or research result. In short, the rich and fruitful discussion concerning the role of values in science (see e.g., Douglas, 2009, Steel, 2010, Hicks, 2014, Brown, 2013, Longino, 1990) and other notions of objectivity inspired by it (see e.g., Douglas, 2009, Longino, 2004) are not directly applicable to our problem.^7^

A detailed discussion of the practical usability of other notions of objectivity presented in literature is beyond the scope of our paper. However, we expect that this cursory sketch provides an overview of the problems with using these notions and motivates the value of a new conceptualization of objectivity of scientific practice.

## To see it from the other side: problems in science and the via-negativa approach to objectivity

There is no generally accepted positive definition of ‘health’ in health care and the medical sciences.^8^ Fortunately, this does not prevent doctors from healing ailments and researchers from developing new drugs and technologies. A positive definition of health is unnecessary, when the instances that reduce or endanger health can be defined and addressed. In brief, health is what remains when the particular infirmities are removed.^9^ Health care and medical science appears to be successful, even in the face of changing definitions, diagnostics, and disagreements about ailments.^10^ We believe that this via-negativa approach can also be applied to the concept of scientific objectivity.

Our negative approach resembles other negative proposals in philosophy (e.g., Koskinen, 2020, Daston & Galison, 2010). Just like these approaches, we conceptualize objectivity as what remains in the absence of certain factors, however, our aim and identified factors are different. Specifically, the purpose of our notion is to be testable and practicable by scientists. Hence, we base our conceptualization on empirical research. We postulate that non-objectivity consists of factors that have been empirically or methodologically identified as making scientific practice susceptible to the actions and decisions of scientist which can inadvertently or intentionally influence research results. Such practices have the propensity to reduce reliability, validity, and replication rates of the results (e.g., Simmons et al., 2011). We assert that the factors constituting non-objectivity translate to a conceptualization of objectivity, which not only preserves some of our intuitions about objectivity but also and more importantly, can be put into practice by scientists (Section 4.2).

The general sense of how the objectivity of scientific practice can be compromised is as follows. Researchers make certain decisions when they design their study and collect, process, and analyze their data. The possibility of choosing between two or more options in these instances are called *researchers’ degrees of freedom* (Simmons et al., 2011; Wicherts et al., 2016). The misuse of which can result in biased^11^ and/or irreproducible outcomes. Kinds of such misuse are identified, for instance, by questioning scientists on their behavior and the behavior of others (e.g., Kerr, 1998, John et al., 2012) or case studies (e.g., Schimmack, 2020) in comparison to data simulation or principles of statistical analysis. As an example of misuse identification, Schimmack (2020) reanalyzed the data of Bem’s *Feeling the Future* experiments (Bem, 2011) and uncovered that effects of precognition were very large if only the data of the first few included participants were analyzed, but decreased raptly to just above the statistical significance threshold towards the end of participant inclusion. Schimmack showed that such a pattern is produced by starting many studies on a non-existing effect and discontinuing all but those that show ‘promise’ (i.e., an initial strong positive effect).

The ways in which scientists can misuse this freedom can be grouped into two categories. Firstly, a scientist can make a priori decisions concerning the research design and data collection, which can preclude certain outcomes or make them more/less likely (i.e. introduce systematic bias in a certain direction). Secondly, a scientist has to make decisions on how to process and analyze the data, which allows her to try all possible combinations of decisions until a positive/desired result is found. In this section, we first focus on problematic practices taking place before or during conducting the study (Section 3.1). Then, we discuss problematic data management and analysis practices (Section 3.2). The section is concluded with a testable conceptualization of objectivity as resilience to such problematic practices.

### Problems before and during research: design, data collection, and measurements

During the early stages of a scientific experiment (e.g., designing the observational study or experiment, sampling, measurement, etc.), a scientist has to make several decisions, which could influence the final result. In some cases, a scientist might make such choices with the aim of obtaining a specific result in mind. Such decisions introduce bias (e.g., Fanelli et al., 2017).

In science, biased research seems to results from the influence of beliefs or prejudices of the scientist on her methodological decisions. For example, scientists make methodological choices that increase the likelihood of getting results that align with the preferences of those that provide the research funds (this is known as ’funding bias’ Nelson, 2014, Jones & Sugden, 2001). Similarly, a scientist can adjust the design of her experiment or observational study, consciously or subconsciously, in order to increase the probability that the results will support her prior beliefs. Typically, biased outcomes(s) only require(s) a single decision or a small number of decisions during the experiment design phase of the research. Taking pharmaceutical research as an illustration, positive results for tested medication is boosted by, for instance, selecting only unrepresentative ‘ideal’ patients, comparing the drug to an ineffective alternative, or using different effective doses for the treatment and control group (e.g., Rothwell, 2005, Travers et al., 2007, Safer, 2002). As another example, the biased studies presented in Wilholt (2008) involve scientists choosing a specific strain of experimental animals, which made the experiments significantly less likely to show the toxicity of the tested substance (in line with the preference of the funding institution). Next to sample selection, bias can be introduced through many of the other decision that a scientist has to make when designing and conducting research, specifically:

1. Which measurement (outcome measure) to use?
2. Which kind of independent variable (experimental manipulation) to use?
3. Which sample to select and how?
4. Setting of the experiment or observational study (when and where)?
5. How and to what extent do researcher and research subject interact?
6. How to perform the measurement (e.g., blinded or unblinded)?

Recognition of features that can introduce bias is reflected in proposals concerning how to counter it. For example, Wilholt (2008) proposed establishing conventions which regulate the way scientist should conduct their studies as a remedy to funding bias. In the case of choosing insensitive animals, he proposed to adopt the following convention:

Because of clear species and strain differences in sensitivity, animal model selection should be based on responsiveness to endocrine active agents of concern (i.e. responsive to positive controls), not on convenience and familiarity. (US Department of Health and Human Services, 2001, p.vii)

Different conventions are and can be implemented in order to impose methodological restrictions on scientists. Some of them force scientists to measure the direct outcome of interest instead of a proxy, use standardized tests or measurements, use random sampling from the population, use random allocations of participants to conditions, use equal group treatment, use blind or double blind design (experimental studies), and/or use data collectors that are blind to the research aim (observational studies). All of these conventions restrict the range of biasing decisions a scientist can make. In addition, these conventions can be empirically tested with respect to prohibiting potentially biasing actions by the scientists and reducing bias in research outcomes.

### Problems after experiments or observations: data management, analysis specification, and result reporting

After a researcher has run the experiment and the data have been collected, several decisions have to be made. For instance, the data need to be processed (e.g., removing outliers, combining variables, binning variable values, etc.), the statistical model needs to be specified (e.g., linear model, multilevel model, structural equation model, etc.), and finally the dependent and predictor variables for the model need to be selected. The assumption is that for each step only one (and the most appropriate) of the possible options is selected. However, research has shown that the general rate of false-positive results^12^ is increased when, instead of taking a single option for each step, several possible combinations of options are explored and only the combinations that culminate in positive results are reported (e.g., Simmons et al., 2011, Wicherts et al., 2016, John et al., 2012, Szucs, 2016). These behind-the-scenes practices that covertly influence results go by the name of *questionable research practices*. The causes of these practices may be the scientists’ (sub)conscious beliefs or preferences, the ambiguity or ignorance about how the methods works and what the statistics are/mean, or the desire to find/see associations and structure in what is being studied. Concretely, at least the following decisions need to be made by a researchers when dealing with quantitative data and performing statistical analyses (this incomplete list is adapted from: Bakker et al., 2012, Nelson et al., 2018, Simmons et al., 2011, Wicherts et al., 2016, Kass et al., 2016):

1. How to handle incomplete or missing data?
2. How to pre-processes data (e.g., cleaning, normalizing, etc.)?
3. How to process data, deal with violations of statistical assumptions (e.g., normality, homoscedasticity, etc.)?
4. How to deal with outliers?
5. Which measured construct to select as primary outcome?
6. Which variable to select as dependent variable out of several that measure the same construct?
7. How to score, bin, recode the chosen dependent variable?
8. Which variables to select as predictors out of the set of measured variables?
9. How to recode or restructure these predictors (e.g., combining variables, combining levels of a variable, etc.)?
10. If and which variables to additionally include as covariates, mediators, or moderators?
11. Which statistical model to use?
12. Which estimation method and computation of standard errors to use?
13. If and which correction for multiple testing to use?
14. Which inference criteria to use (e.g., p-values and alpha level, Bayes factor, etc.)?

Note that, if such decisions needs to be made and how many option the scientists has to choose from depends on how the study was designed and the structure and amount of data that were collected.

Currently, there are already some potential strategies for restricting uses of questionable research practices (i.e., ad hoc decision making in order to get positive results, also known as p-hacking). For instance, a) preregistration of the study from design to analysis (e.g., Chambers, 2013, Wicherts et al., 2016, Nosek et al., 2018); b) data and analysis blinding (e.g., MacCoun and Perlmutter, 2015); and c) running several/all of the (theoretically) possible tests in a *multiverse analysis* (Steegen et al., 2016).^13^ The effectiveness of these strategies can be empirically verified by researching their effects in, for instance, replication studies. It should be noted that such strategies are not mutually exclusive and that combinations are possible, because they all restrict researcher’s degrees of freedom without introducing new ones. For instance, not all decisions can be made in advance, precluding their preregistration. In such a case, some of these can be caught by data blinding, because the scientist might not know what the data will look like in advance, though has an analysis plan that can be communicated for the independent data analysis. In addition, the multiverse analysis can be employed for those elements of the research that have an exploratory nature that do not allow for data blinding and handing the analysis to someone else.

### To Sum up: a conceptualization of objectivity

Our negative version of conceptualizing objectivity ties it to scientific problems that result from the decisions and actions of individual scientists. These problems are notoriously hard to detect. For instance, a report of a study during which questionable research practices were used can be indistinguishable from a report of a study where scientists actively tried to avoid influencing the results. If objectivity is just absence of these problems, then testing it is extremely difficult to impossible. On the other hand, we can easily tell if precautions against such problems (e.g., preregistration) are present and thus how resilient a given practice is. Therefore, we state that a scientific practice becomes more objective when it becomes demonstrably more resilient to actions and decisions that have the potential to influence its outcome; concretely, when:

- the study design and data collection becomes demonstrably more resilient to the scientists’ influence on the data;
- and the data processing and analysis become demonstrably more resilient to ad hoc decision making and selective reporting of positive results.

In the limit, a practice is objective when it is impervious to biasing influences and precludes ad hoc decisions and actions.

Our approach has two clear advantages. 1) It is empirically verifiable. 2) It does not require universally agreement about factors that reduce objectivity nor does the procedure for identifying these factors need to be objective. Our notion, in opposition to traditional conceptualization (e.g., value-free objectivity), ties objectivity to features of scientific practice, the existence of which can be empirically tested (e.g., was the study preregistered or not).^14^ These features can, for instance, be collected in a form of a checklist (see the Appendix for a first setup). Concretely, such a checklist could in principle be used by reviewers to evaluate submitted manuscripts on the precautions taken against biasing influences; or by readers of published papers who want to assess their trustworthiness; or by reviewers (writers) of grant applications to evaluate (show) that future results will be as insulated as possible against biasing effects.^15^ In addition, objectivity according to this conceptualization can be verified by assessing the extent of systematic bias and inflated false-positive rates in a body of literature. The presence of objectivity promoting features like preregistration decreases the chance of a given study being a false positive. Therefore we can indirectly test the objectivity of studies, for instance, by testing consistency of results between preregistered experiments in comparison to consistency of results between non-preregistered experiments.

The second advantage follows from the first. We do not claim that the list of objectivity reducing factors on which our conceptualization is based is exhaustive. Moreover, some factors might be considered controversial as objectivity reducing or it may not be objective how factors are included, while other are not. This is not problematic for our proposal, because a) the identification and inclusion of factors is based on robust empirical results and methodological considerations; and b) their impact on the quality of the study, as explained in the previous paragraph, can be empirically verified.

## Discussion

In this paper, we have offered a novel and practicable conceptualization of scientific objectivity. We have argued that many of the popular philosophical attempts at defining objectivity are not practicable and are likely to be impossible to implement by individual scientists. As we have argued, some of the theories aim at reconstructing the way the philosophers or scientists understand objectivity rather than proposing a normatively compelling notion. Secondly, some of the normative proposals define objectivity in terms of features that are prohibitively difficult to test empirically and hence use in practice. For example, testing conceptualizations that define objectivity in terms of the intentions of scientists, like value-free objectivity, would require real-time access to the mind of scientists during research.

In our approach, we have used findings from empirical research and methodological considerations to identify features of scientific practice considered to be problematic (i.e., potential causes of bias and inflated false-positive rates). We postulate that resilience to these features constitute objectivity. Given these features, scientific practice approaches objectivity when it becomes less vulnerable to decisions and actions of scientists that can influence its outcome.

In this section, we discuss the limitations and implications of our conceptualization. In the appendix, we present a draft for a tool that can be used to assess the objectivity of scientific endeavours (e.g., published papers, submitted manuscripts, proposed research in grant applications, etc.). In addition, we suggest investigations into a tool such as ours to test and improve its validity and reliability. We close this paper with a detailed illustration of how such a tool could be usefully implemented.

### Limitations

*Incompleteness*. Plausibly, in our paper we do not reach a complete list of ways in which scientific practice can be compromised. Therefore, it is most likely that we did not reach a complete definition of objectivity, though rather a number of currently identified necessary conditions. However, our approach does provide a framework for learning from empirical research and methodological developments when, where, and how particular factors compromise scientific objectivity. Even with this limitation, we believe that our conceptualization is an improvement over previous attempts of conceptualizing objectivity and can still be used in a fruitful way (Sections 3.3 and 4.2).

*Ritualization*. Some might argue that restricting researchers in the proposed way will actually reduce objectivity. For instance, the (faulty) use of the Null Hypothesis Significance Testing procedure (NHST) has been described as a restrictive ritual; a practice that discourages informed reasoning and prescribes certain actions and decisions. The NHST ritual has been considered to be the main cause of the inflated number of false-positive results in science (Gigerenzer, 2004; Stark & Saltelli, 2018; Ioannidis, 2005), which is the opposite of what an objective method should achieve. However, the NHST ritual only appears to restrict researchers and provides just the illusion of objectivity. In particular, apart from inference criterion (i.e., an observed statistic lower than a conventional threshold), this ritual does not restrict (mis)use of degrees of freedom (mentioned in Section 3.2) at any point during the research process. Specifically, and in contrast to recommendations of our proposal, ad hoc decision-making in data management, analysis, and result reporting are not prohibited in the NHST ritual. It might even be considered that this partial formalization enshrines a false sense of objectivity that is actually harmful to the quality of scientific results (e.g., Gigerenzer, 2004, Simmons et al., 2011). In other words, if the ritual had been restrictive in ruling out questionable research practices, it would actually promote objectivity. Our conceptualization does recommend these additional restrictions. Also, in contrast to the conservative nature of a ritual, our conceptualization is (meant to be) adaptive; developed in accordance with novel discoveries concerning problematic scientific practices and methodological changes in science.

*Restricted*. Our conceptualization is restricted to practice of quantifiable or countable research, which precludes qualitative research and non-empirical practices. Qualitative research is currently omitted from our definition, because, to our knowledge, empirical research and methodological considerations on the particulars of systematic bias and false-positive rate inflation in the use of qualitative methods are currently absent in the academic literature. It remains an open question if our or a analogous notion can be applied to qualitative research.

*Scientist-independent problems*. In some cases, a source of negative influence on research results is independent of the decisions of a scientist (e.g., Biddle, 2007, Bueter, 2015, Harding, 2015, Leuschner, 2012, Longino, 1990). For example, a scientist may be restricted in access to particular instruments, samples, or treatments of research subjects for external reasons (e.g. ethical, political, financial, practical, etc.). Therefore, the results can be compromised, though not because the scientist misused degrees of freedom. It is also possible that some internal features of a research field or used methodology cause the results to be systematically biased. In such a case, the culture and conventions of a particular area of research may restrict individual scientists to particular measurement instruments and research subjects, which could produce spurious and biased findings. For instance, culture and politics can influence which research projects get funded and thus carried out (e.g., Bueter, 2015, Elliott & McKaughan, 2009). These factors might also influence which research results get published (i.e., publication bias). Specifically, at the moment it seems that most scientific journals prefer to publish articles describing experiments with positive results and/or scientists submit only positive results to these journals. This bias against negative results precludes some research from entering the scientific literature, which inflates the rate of published false-positive results. Consequently, even if the scientific practice of each individual scientist is (as) objective (as possible), the false-positive rate will still be inflated to an unknown degree. Publication bias (e.g., Malički & Marušić, 2014) and other similar scientist-independent problems (e.g., Leuschner, 2012, Biddle, 2007) are discussed extensively in the literature and some solutions were proposed (see e.g., Carroll et al., 2017, Longino, 1990, Harding, 2015). These problems are larger than the individual scientists and thus the proposed solutions typically involve changing the social arrangement of science rather than practices and procedures used by individual scientists. For example, (Biddle, 2007) proposes to implement a system of institutionalized criticism to counter the corrupting effect of financial stakes on the integrity of research, another major scientist-independent problem. However, the two types of problems are distinct and therefore they require different solutions. Misuse of degrees of freedom requires the improvement in objectivity as understood in a way we have described above. On the other hand, external limitation requires improvement in the social structure of science and possibly general improvements in scientific methodology. Thus, we acknowledge the existence of these social, cultural, political, and technical problems and are in favor of programs addressing these issues. Additionally, the solutions on both social and individual level problems are complementary and might be combined into a more complete proposal.

*Exploratory research and serendipitous discoveries*. Many (if not most of the) famous scientific breakthroughs have been serendipitous discoveries. These discoveries were most likely the product of exploratory research that were neither done by unbiased scientist nor completely free from practices that would now be labeled as ’questionable’. It should be noted that we do not object to these practices and even see them as a vital part of science. However, when it comes to verifying these findings and integrating them in the rest of science, we firmly believe that these discoveries should be tested with a practice that is as objective as possible.

*Too demanding.* Clearly, our conceptualization is very exacting. Not many or maybe even no scientific practice, past or present, is objective in this sense. This is a criticism that has also been leveled at procedural objectivity (Jukola, 2017).^16^ Be that as it may, this does not prevent our notion from being useful. As we will demonstrate in the next section (Section 4.2) we can compare the relative objectivity of two methods even if neither of them is fully objective according to our conceptualization. Secondly, the notions give us a clear idea of which modifications of a given practice increase its objectivity. In light of this, we believe that the usefulness of our conceptualization is not impaired because it is hard to satisfy. It is something to strive for, not necessarily something to reach.

*Objective research does not guarantee true nor trustworthy results*. Even if the work of a scientist did not suffer from anything that could jeopardize the research’s objectivity, it is still possible that the results are not true (i.e., do not reflect or represent reality). It could be as innocent as a false-positive or it might be that the measurement instrument is not adequate for investigating the phenomenon at hand. Either way, we should be clear that objectivity of a practice cannot be equated with scientific truth generation. Similarly, even when scientific practice is (as close to) objective (as possible), it may still suffer from low reliability (i.e., noisy measurement) or lacks validity (i.e., does not measure what it is supposed to measure). In other words, validity and reliability might be necessary to guarantee the quality and trustworthiness of results. Furthermore, the possibility of trustworthy results without *procedural objectivity* has been leveled as a criticism against this type of objectivity (Jukola, 2017). However, according to our conceptualization, perfect/high reliability, validity and thus trustworthiness are neither necessary nor sufficient conditions for the objectivity of the scientific practice that produced the results. That being said, we should still care about objectivity, because validity and reliability are promoted by it (Section 4.2).

### Implications and applications

The primary advantage of our approach to objectivity is that, in contrast to traditional theories of objectivity, it can be applied in science. For instance, our notion can be used to assess and address currently salient problems in science (i.e., the replication crisis: Harris, 2017) and evaluate suggested solutions to problematic scientific practices. Concretely, our conceptualization of objectivity can be captured in an tool that can be tested and calibrated (for an example, see Appendix A).

Increasing objectivity of scientific methods is a necessary step in remedying problems, such as the replication crisis (e.g., Harris, 2017). This crisis is constituted by the fact that results from many scientific experiments are not reproduced in replication studies (for a discussion see: Open Science Collaboration, 2015, Romero, 2016). Concretely, that experiments with similar or identical designs conducted by different scientists (or by the same researchers for the second time) delivered widely different results. The exact percentage of replicablility is unknown, though some indication might be gleaned from large scale replication projects (e.g., Open Science Collaboration, 2015, Klein et al., 2018). In the case of the Open Science Collaboration (2015), hundreds of scientists collaborated to attempt replication of one-hundred experiments published in prestigious psychological journals. Less than half of the attempts were successful;^17^ clearly a disappointing result.

Replicability can be compromised by many factors. One of them is the misuse of degrees of freedom (e.g. Simmons et al., 2011, Wicherts et al., 2016). Specifically, biased studies are more likely to deliver results which fit the particular interest of the scientist (Section 3.1), or general interest in positive results or absence of negative results (Section 3.2), which therefore will likely disagree with the results of unbiased experiments; decreasing the overall replicability. Now, if the objectivity of scientific practice (i.e., resistance against bias and questionable research practices) is increased, then replicability on any reasonable metric will increase. In light of that, increasing objectivity seems to be a necessary steps toward solving the replication crisis and its effectiveness will be clearly observable in the published scientific literature.

In addition, our notion gives clear indications of which suggested solution to problematic scientific practices will most likely be successful. Some of these restrict scientists directly (e.g., preregistration requirement, random sampling, randomization, etc.), while others make it harder to exploit degrees of freedom (e.g. blind analysis). Because of that, they improve the objectivity to a certain extent. On the other hand, for some of the proposals it is not clear if they are capable of improving objectivity. The *Reformist Package* is an example of such a proposal. It requires that the first author of a paper on a scientific experiment states all potential conflicts of interest. This amounts to explicitly listing all sources of funding that supported his/her work and claiming full responsibility for the result and decision to publish it. The Reformist Package has some proponents in scientific literature (e.g., Stelfox et al., 1998) and some of the most important scientific journals (e.g., Lancet, Journal of the American Medical Association, etc.) adopted it in their publishing policy. However, according to our conceptualization, it is not clear at all if the proposal improves the objectivity. The Package is forcing scientists to reveal potential causes of systematic bias in the form of financial ties, but it does not safeguard the experiment against actions that can introduce this bias. Our conceptualization predicts that the Reformist Package is ineffective in dealing with the influence funding agencies have, via their researchers, on the results. This is corroborated by the dissatisfaction concerning its ineffectiveness common in current literature (e.g., Schafer, 2004), and is supported by the results of empirical research (e.g., Cain et al., 2005).

Additionally, our notion can be used to assess the objectivity of research practices reported in scientific papers (e.g., through a checklist; see Appendix A). As an example, we can use the previously mentioned, notorious precognition paper by (Bem, 2011). This article reports nine experiments that allegedly provide evidence for the hypothesis that future events affect human beliefs (precognition). These results are treated by scientists with skepticism because the existence of precognition is inconsistent with laws of nature (e.g., the second law of thermodynamics), common sense, and everyday experience. Not surprisingly, the subsequent replication attempt failed (e.g., Ritchie et al., 2012, Galak et al., 2012) and evidence of the use of QRPs was found (e.g., Schimmack, 2012, Francis, 2014, Schimmack, 2020).

The procedures employed for the nine experiments would not score high on our conception of objectivity. Some aspects of the design of the experiment promote objectivity, the outcome measure and intervention are directly connected to the studied phenomenon and the allocation of subjects was random. On the flip side, participants were exclusively psychology students, the study was not preregistered, and neither the blind analysis nor multiverse analysis was used.^18^ The absence of such countermeasures makes the experiment susceptible to the QRPs. An example of such a practice is looking at the initial data of many started experiments and continuing only those that look ’promising’; i.e., only continue with studies that show high initial effect sizes that are due to random chance alone (for evidence for this claim, see Schimmack, 2020).

This is an intuitive result given the skepticism concerning the results of precognition. Moreover, as we have seen, the subsequent replication failed to replicate the original result and evidence suggesting the the QRP were used during the experiment. The objectivity of many other older experiments will be similarly disappointing. The methodological problems central to our conceptualization of objectivity were not widely acknowledged and the countermeasures against them were rarely implemented. This may seem to be a disappointing consequence but it is consistent with low rates of replicability of classical studies (e.g., Klein et al., 2018) and acknowledges the recent rapid development in scientific methodology.

Finally, our conceptualization of objectivity is compatible with, and follows the spirit of many traditional theories of objectivity. Our notion is based on the intuition that objectivity is essentially about minimizing the influences that the individual traits of a scientist have on her research (results). This intuition inspired many other conceptualizations of objectivity, for instance, value-free objectivity, procedural objectivity or Koskinen’s theory (Section 2). Specifically, the value-free conception of objectivity claims that a scientific justification is objective as long as it is not influenced by non-epistemic values. However and in contrast to our conception, the value-free objectivity is hard to assess and therefore use in practice. This is the case because there is no reliable way to assess and test what was the motivation behind any methodological choice.

The same goes for procedural objectivity. This proposal has been previously criticized in Jukola (2017) and we identify two additional problems. First, as in case of VFI, it is prohibitively difficult to verify if a given process is objective in this sense. Secondly, the conceptualization is too restrictive. For example, when statistical methods are used to analyze data it is always the case that the result of an experiment will be different when conducted second time at some level of precision. Furthermore, there is always the possibility of false-positives and false-negatives. Therefore, it seems that no such study can be objective in the sense of the procedural objectivity. Our conceptualization does not suffers from those two difficulties.

Another feature that distinguishes our conceptualization is that it explicitly requires the scientific procedure in question to be demonstrably resilient to problematic practices rather than just free of them. This makes our conceptualization testable (as the presence of the countermeasures is evident in contrast to the presence of the problems) and distinguishes it from other proposals based on similar intuitions. For example, the conceptualization of objectivity as minimizing epistemic risks which arise from the imperfections of epistemic agents from (Koskinen, 2020) does no include such an external transparency requirement. Under such a conceptualization, a given scientific procedure could objective, but this would be inaccessible to anybody (e.g., a reviewer of an article) except the responsible scientist. Our theory does not suffer from this problem.

Furthermore, our notion is consistent with all descriptive theories, because we do not claim anything about how the concept is used and understood by scientists or natural language users. Besides, some of these descriptive conceptualizations seem to be based on the above mentioned intuition as well. For example, the epistemic risk account of objectivity of (Koskinen, 2020), seems to be similar in spirit to our proposal. It claims that objectivity consists in averting epistemic risks arising from imperfections of epistemic agents. Adhering to the recommendations of our proposal averts some of such risks, for example, the risk of delivering a biased result due to study design choices (Section 3.1). In other words, her description of how objectivity is understood fits to a certain extent with our recommendations. Regulatory objectivity, described in (Cambrosio et al., 2006), is another example of a descriptive conceptualization based on the same intuition. It is built on the historical analysis of objectivity from (Daston & Galison, 1992, 2010). Regulatory objectivity consists of conventions which aim to ensure research quality, specifically:

*Regulatory objectivity*, that is based on the systematic recourse to the collective production of evidence. Unlike forms of objectivity that emerged in earlier eras, regulatory objectivity consistently results in the production of conventions, sometimes tacit and unintentional but most often arrived at through concerted programs of action. (Cambrosio et al., 2006, p.1)

Recent developments are interpreted as the emergence of a new type of objectivity. Implementing and developing such conventions fit our recommendations for the prevention of methodological choices that can bias results or inflate false-positive rates. Again, there is coherence between our normative proposal and the descriptive theory which describes how scientists understand the objectivity.

### Conclusions

Let us once again imagine our scientists, Dr. Jane Summers. Dr. Summers is starting a new experiment (e.g., the effects of caffeine on attention, short-term memory, and long-term memory in psychologically healthy adults) but this time she has a grasp on the notion of objectivity and will include (some of) the objectivity promoting precautions. Specifically, when she designs the study, she ensures that for all intents and purposes the participants selection is random from the population of interest (e.g., males and females, age 21 and up that do not suffer from psychological disorders) and that the non-response rate is not biased (e.g., equal non-response in age and gender), that the measurement instruments come with published validation (i.e., standardized test for attention and memory), the participants’ allocation to conditions (e.g., coffee with a high dose of caffeine or decaffeinated coffee) is random, and the experiment is double-blinded (i.e., both participant and experimenter are unaware of experiment condition and purpose). Dr. Summers preregisters the study design and the analysis (e.g., structural equation model) of the main effect of interest (e.g., caffeine positively affects long-term memory, mediated by attention and short-term memory). She will have her data blinded and processed by an independent researcher. In addition, she reserves a room for a multiverse analysis. In Dr. Summers’ case, not much is known about the complex relation between dependent and independent variables and its mediation or moderation by participant characteristics (e.g., sex, age, daily caffeine consumption, etc.). Thus, apart from the main model suggested by theory and previous research, she wishes to explore other theoretically possible options. Specifically, she performs and reports the results of the analyses of all theoretically possible models and summarizes their results in a multiverse analysis. By taking these steps, Dr. Summers restricts many ways in which her study can be biased and thereby improves the objectivity of her work.

Similar steps may be taken in order to improve the objectivity of Bem’s (2011) experiments. The main problem with the experiments is the (possible) use of QRPs. In particular, he seemed to have started many experiments and only continued collecting data on those that showed ‘promising’ results (Schimmack, 2020). This could be countered by ensuring preregistration of all initial studies and requiring an appropriate analysis plan if Bem intended to apply sequential analyses. If the diagnosis by Schimmack (2020) is correct, this alone would significantly increase the reliability of the experiments to the point that it would be highly improbable that they would deliver the suspicious result. In addition, one could require a multiverse analysis over control variables (e.g., gender) and experimental variation (e.g., subcategories of stimuli). As an example for such a requirement, in one of the experiments the precognition effect was observed for pornographic stimuli, but not for neutral stimuli (Bem, 2011). In brief, it is to be expected that implementing the safeguards proposed in this paper would have prevented Bem from getting his results that humans have the ability to feel the future.

To summarize, in this paper we have presented a practicable notion of scientific objectivity. In our opinion, popular disquisitions on objectivity are focused on what the concept means and how it is used, but they do not provide scientists with any guidance on how to improve or assess the objectivity of their work. We presented our empirically informed version of via negativa approach to objectivity and conceptualization of objectivity as methodological resilience. Finally, we showed that and how this new conceptualization can plausibly be used by scientists. In the present form, our theory is far from perfect or complete. At the same time, like science itself, it has the potential to be adjusted and developed to move ever closer to adequacy and completeness.

### Declarations

The authors have contributed equally to the manuscript. There are no conflicts of interest. The research was supported by Starting Investigator Grant No. 640638 (“OBJECTIVITY—Making Scientific Inferences More Objective”) of the European Research Council (ERC).

## Appendix A: Setup of a checklist for objectivity assessment

Given the negative nature of our notion, our checklist consists of questions assessing how susceptible a study is to suffer from the mentioned problematic practices. Concretely, a checklist consists of yes-no questions that indicate the presence or absence of features that prevent problematic practices.

Questions concerning the study being bias-resilient:^19^

1. Was (were) the outcome measure(s) directly related to the phenomenon of interest as stated in the research aim or research question? (e.g., ‘death rate’ to ‘death by cardiac arrest’)
2. Was (were) the intervention(s) clearly related to phenomenon of interest as stated in the research aim or research question? (e.g., ‘cardiac arrest reducing medication’ to ‘death by cardiac arrest’)
3. Was sampling procedure random?
4. Was the sampling procedure capable of producing a sample representative of the population? (i.e., do inclusion/exclusion criteria allow all member of the population)
5. When the subjects are volunteers, was the (non-)response rate similar across participant characteristics? (e.g., equal between men and women)
6. Was the allocation of the subjects to the experiment conditions random?
7. Were both the experimenter and the subjects blind to the experiment condition?
8. Was the drop-out rate of subjects similar across the experiment conditions?
9. If any answer to these questions was ’no’, were proper steps taken to ameliorate the potential bias that could have resulted from it?

Questions concerning the study being resilient against bias and false-positive rate inflation due to questionable research practices:

1. Was the study preregistered?
2. If so, was the following specified in the preregistration:
  - Management of missing and incomplete data.
  - Pre-processing of data (e.g., how to clean and normalize).
  - Data processing and dealing with violation of statistical assumptions.
  - Management of outliers.
  - Statistical analysis/model.
  - Dependent variable(s) of the model.
  - Predictors/covariates of the model.
  - Estimation method and computation of standard errors.
  - Inference criteria.
3. If preregistered, did the final report conform to this preregistration? Specifically, did the final report conform to the preregistration on:
  - Management of missing and incomplete data.
  - Pre-processing of data (e.g., how to clean and normalize).
  - Data processing and dealing with violation of statistical assumptions.
  - Management of outliers.
  - Statistical analysis/model.
  - Dependent variable(s) of the model.
  - Predictors/covariates of the model.
  - Estimation method and computation of standard errors.
  - Inference criteria.
4. If the final report did not completely conform to the preregistration, was the particular deviation handled by blinding data or blinded analysis?
5. If the final report did not completely conform to the preregistration, was the particular deviation handled by using a multiverse analysis and reporting all (theoretically) possible ways of handling this case?
6. If the study was not preregistered, was blinded data management and analysis used?
7. If blinded data management and analysis was used, was it used on the following:
  - Management of missing and incomplete data.
  - Pre-processing of data (e.g., how to clean and normalize).
  - Data processing and dealing with violation of statistical assumptions.
  - Management of outliers.
  - Statistical analysis/model.
  - Dependent variable(s) of the model.
  - Predictors/covariates of the model.
  - Estimation method and computation of standard errors.
  - Inference criteria.
8. If the study was not preregistered and the data management and analysis was not blinded, was a type of multiverse analysis performed?
9. If a type of multiverse analysis was performed, did it incorporate the following elements:
  - Management of missing and incomplete data.
  - Pre-processing of data (e.g., how to clean and normalize).
  - Data processing and dealing with violation of statistical assumptions.
  - Management of outliers.
  - Statistical analysis/model.
  - Dependent variable(s) of the model.
  - Predictors/covariates of the model.
  - Estimation method and computation of standard errors.
  - Inference criteria.

It needs to be noted that such a tool needs to be further developed, tested, and calibrated. To be useful, this objectivity checklist should of course be (to a large extent) reliable and valid. In the first case, inter-rater reliability and intra-rater reliability should be assessed. I.e., have different subjects use the tool and measure the agreement between their results (Cohen, 1960; Fleiss, 1971), and have subjects use the tool on two or more different occasions on the same material and measure the similarity of results between these occasions (Gwet, 2008). Close similarity between scores indicate that users will in general give similar scores when using the checklist. If there are questions in the checklist that score low on inter-rater and/or intra-rater reliability, then they should be rephrased or dropped. The validity of the checklist can be assessed by empirically verifying if research that scores high on the checklist are less prone to produce problematic results (e.g., have a higher replication rate) in comparison to research that score low on the checklist (i.e., criterion validity). For now, we do not have a scoring system of the checklist. The easiest scoring system would be to use the proportion of ’yes’ answers of the total number of questions that are relevant for the report that is evaluated. However, this scoring system should be further developed and tested. Also, scientific practice could be simulated to assess to what extent bias and false-positive rate inflation could still be introduced with varying levels of objectivity according to the checklist. Results from such simulation studies could also be used to calibrate the scoring system. Finally, scientists could be enlisted to perform mock research to attempt to bias outcomes and/or produce false-positive results with varying levels of objectivity safeguards in play. These mock studies might identify weaknesses and gaps in the tool (and objectivity conceptualization), which could be used when calibrating the tool and supplementing/removing elements.

## Footnotes

1 Dr. Summers is a fictional character, though we postulate that she is representative of current practitioners who work on the improvement of research practice and her considerations of potential problems result from a synthesis of methods textbooks that have recently fallen from grace for recommending questionable practice.

2 A similar description of objectivity can be found on Wikipedia (2019).

3 Interestingly, there are not many references on the importance of objectivity for science. Scientists we spoke to consider its relevance obvious and self-explanatory to such an extent that it does not warrant explicit explanation and justification.

4 For reasons clarified in Sections 3 and 4, we restrict our definition to research that works with non-qualitative (quantitative or countable) data.

5 We address this limitation in more detail in Section 4.

6 Despite this descriptive goal, there are some normative remarks in the paper. For example, Douglas refers to her earlier work (Douglas, 2000) in which she argued that one of the conceptualizations of objectivity, value-free objectivity, is impossible to realize in practice. However, this does not detract from the main aim.

7 One may wonder how the discussed descriptive theories would do if put in the normative role. We believe that the theories would face similar difficulties. The theories from (Douglas, 2004) and Koskinen (2020) seems to be too general to be useful in their present formulation. Douglas’ theory is composed of many individual notions all of which generate different predictions. It is not supplemented with a rule describing which notion to use in each individual case. Similarly, if we translate Koskinen’s theory into methodological advice, something like, a scientific procedure is objective to the degree to which it minimizes epistemic risks which arise from imperfections of scientists, it seems too general to be useful. At the same time, we believe that a concretization of Koskinen’s theory could lead to a plausible normative proposal, in a very general sense similar to our proposal. These observations do not constitute objection against the theories, they were proposed as descriptions, and criticizing their performance beyond their original purpose is not a fair criticism.

8 The World Health Organization defines health as ‘a state of complete physical, mental and social well-being and not merely the absence of disease or infirmity.’ (World Health Organization, 1948, p.100). Which is essentially a negative definition augmented with an well-being requirement. The definition is considered controversial and with little added benefit over the original negative definition (e.g., Jadad and O’Grady, 2008). Adding ‘well-being’ just kicks the can down the road.

9 In this paper, the negative definition of ‘health’ is used as an analogy to clarify our approach. Our conceptualization has no stake in this definition or its controversies.

10 Some might argue that medical science is not as successful as it purports to be and suffers from diminishing returns (Stegenga, 2018). However, this does not detract from the fact that medical science is successful to at least some degree and the mentioned problems do not necessarily result from a lack of a positive definition of health (see for instance Firestein, 2015).

11 For clarification, bias, as discussed in this paper, is used to indicate systematic error introduced by behavior of the scientist (e.g., Ioannidis, 2005). This is different from bias in psychology (e.g., MacCoun, 1998) where it is used to classify cognitive heuristics (e.g., confirmation bias, bandwagon effect, anchoring, etc.). These heuristics might indirectly influence results, but these distant causes are irrelevant to our approach.

12 No matter how precise an instrument is and no matter how stringent the evidence requirements are, there is always a non-zero probability that the result of a study does not reflect reality (e.g., positive outcome of a HIV test when the person is actually HIV negative). Statistical methods of analysis come with certain rules and assumptions, which must be followed in order for this probability to have a known maximum. In other words, if a study is performed according to its rules, none of the assumptions are violated, and it is repeated a large number of times, the proportion of false-positive results (i.e., an effect is observed while actually no effect exists) is at most equal to this probability, which is or can be known.

13 In-depth discussion of these strategies is beyond the scope of this paper. Function, benefits, and limitations of these strategies can be found in the cited papers.

14 Note that it is not the testing of whether or not certain potentially biasing actions were made during the research, but what precautions were in place to preclude such actions.

15 Similar checklists have been developed and are in wide use as tools for assessing methodological quality of studies (e.g., Downs & Black, 1998, Sindhu et al., 1997) when, for instance, appraised for inclusion into a systematic review (e.g., Haidich, 2010). Recently, a checklist to assess scientific transparency published (Aczel et al., 2020) that awaits application.

16 Procedural objectivity is the claim that there is objectivity when different scientists using the same procedure get the same/similar results (e.g., Porter, 1995). This is one of the notions included in Douglas’ (2004) pluralist account of objectivity.

17 A clear and formal definitions of replication is still absent and several benchmarks were used in this paper. On none of them did the replication rate exceed 50%.

18 We acknowledge that these practices were not widely known at the time. However, this does not mean that we cannot assess previous practice by current standards.
